# Increased movement-related signals in both basal ganglia and cerebellar output pathways in two children with dystonia

**DOI:** 10.3389/fneur.2022.989340

**Published:** 2022-09-09

**Authors:** Estefania Hernandez-Martin, Enrique Arguelles, Mark Liker, Aaron Robison, Terence D. Sanger

**Affiliations:** ^1^Department of Electrical Engineering and Computer Science, University of California, Irvine, Irvine, CA, United States; ^2^Neuroscience Institute, Children's Hospital of Orange County (CHOC), Orange, CA, United States; ^3^Department of Neurology, Children's Hospital of Los Angeles (CHLA), Los Angeles, CA, United States; ^4^Department of Neurological Surgery, Keck School of Medicine, University of Southern California (USC), Los Angeles, CA, United States; ^5^Department of Biomedical Engineering, University of Southern California (USC), Los Angeles, CA, United States

**Keywords:** cyclic drawing task, deep brain signals, muscle signals, movement disorders, deep brain stimulation

## Abstract

The contribution of different brain regions to movement abnormalities in children with dystonia is unknown. Three awake subjects undergoing depth electrode implantation for assessments of potential deep brain recording targets performed a rhythmic figure-8 drawing task. Two subjects had dystonia, one was undergoing testing for treatment of Tourette Syndrome and had neither dystonia nor abnormal movements during testing. Movement-related signals were evaluated by determining the magnitude of task-related frequency components. Brain signals were recorded in globus pallidus internus (GPi), the ventral oralis anterior/posterior (VoaVop) and the ventral intermediate (Vim) nuclei of the thalamus. In comparison to the subject without dystonia, both children with dystonia showed increased task-related activity in GPi and Vim. This finding is consistent with a role of both basal ganglia and cerebellar outputs in the pathogenesis of dystonia. Our results further suggest that frequency analysis of brain recordings during cyclic movements may be a useful tool for analysis of the presence of movement-related signals in various brain regions.

## Introduction

The basal ganglia, cerebellum, and thalamus compose a complex system whose combined roles in human motor control remain unknown. The availability of intracranial electrical recordings during surgery for implantation of Deep Brain Stimulation (DBS) electrodes has provided some ability to observe activity in a variety of different disorders, including Parkinson's Disease, Essential Tremor, and Dystonia. Interpretation of this information has been limited by (1) lack of availability of recordings from healthy subjects for comparison, (2) recording from only one or two sites at a time, and (3) lack of techniques that allow separation of movement-related signals from other ongoing brain activity.

Here we report data from up to 60 simultaneously recorded electrodes in globus pallidus internus (GPi), thalamus ventral oralis anterior/posterior nuclei (VoaVop), and thalamus ventral intermediate nucleus (Vim), along with surface electromyography (sEMG). All signals are compared to the kinematics of movement. We use a rhythmic task so that by analysis of the task-related frequencies we can determine the contribution of movement signals in each deep brain region.

We report data from 3 subjects: one with generalized dystonia, one with hemidystonia, and one with Tourette syndrome (TS). The significance of these subjects is that we recorded from the brain hemisphere contralateral to the less affected side of the patient with hemidystonia. This area is not expected to be healthy (indeed, the patient ultimately benefited from DBS electrode implantation in GPi and thalamus on this side). The features of dystonic symptoms for two patients were hypertonic (1). The patient with Tourette syndrome, while not healthy, does not have any difficulty with routine arm movement when not having tics. In fact, during the testing period (approx. 7 days), the patient did not exhibit any tics at all; upon awakening from electrode implantation (2), the patient was noted to have complete cessation of motor and vocal tics prior to electrical stimulation through the electrodes, and thus movement phenotype is normal. Therefore, while the patient with TS cannot be considered a healthy control, he nevertheless provides an example of healthy behavior. While this is a very limited sample, the three patients represent an important range for comparison: (1) active dystonia (generalized dystonia patient), (2) mild dystonia (less affected side of the hemidystonia patient), (3) no dystonia (TS patient).

The cyclic drawing task allows us to determine the relationship between signals from deep structures and the kinematics and electromyography signals. The task involves continuous and repeated Figure 8 movements on a tablet. The particular value of this task is that the desired movement consists of two frequencies: the movement in the direction of the short axis of the Figure 8 has twice the frequency of the movement in the direction of the long axis. This allows us to differentiate contributors to horizontal and vertical movement by examining the frequency components of brain or muscle signals. It also allows us to determine which components of activity are task-unrelated, because frequency components that are at neither the horizontal nor vertical frequency do not contribute to the desired movement and are either irrelevant or represent errors in movement. We have previously shown the potential of the cyclic drawing task to objectively relate muscle activity measured by surface electromyography (sEMG) to the kinematics of movement (3).

We make use of data obtained during targeting for deep brain stimulation (DBS) in children. At our institution, the targeting procedure includes placement of temporary depth electrodes in potential target regions, and children are subsequently evaluated while awake in a regular hospital bed. This allows unconstrained performance of tasks, including reaching or repetitive arm and finger movements. The targets we evaluate for DBS at our institution include ventral oralis anterior/posterior (VoaVop), ventral intermedius (Vim) and ventral anterior (VA) nuclei of the thalamus (4, 5), as well as globus pallidus internus (GPi) (6) and subthalamic nucleus (STN) (7, 8).

We will analyze data from the basal ganglia output (GPi), thalamic targets of this output (VoaVop), and thalamic targets of cerebellar output (Vim) in order to investigate the relative contributions of basal ganglia and cerebellar outputs to dystonic movement.

## Materials and methods

### Subjects

Subjects were diagnosed by a pediatric movement disorder specialist (T.D.S.). All patients provided signed informed consent for surgical procedures in accordance with standard hospital practice [Children's Hospital of Orange County (CHOC)]. The patients, or parents of minor patients, also signed an informed consent for the research use of electrophysiological data and Health Insurance Portability and Accountability Act (HIPAA) authorization for the research use of protected health information. Three male patients (Table 1) with movement disorders [hypertonic dystonia; location hemi and generalized (known from here as hemidystonia and generalized dystonia), and Tourette syndrome] executed the motor task while surface electromyography (sEMG) and brain signals were simultaneously recorded. For this study, we analyze data from electrodes in GPi, VoaVop, and Vim. In most cases, electrodes were also placed in other thalamic targets including ventral anterior (VA) or ventral posterolateral (VPL) subnuclei, but those were not shown as part of this study because they were not common targets across the subjects (Supplementary Figure S1).

**Table 1:** Demographic characteristics.

**Patients**	**Etiology**	**Characteristics**	**Gender**	**Age**	**Leads**
NMU1^a^	Dyskinetic CP (vasculitis)	Left dystonia	M	14	GPi; Voa/Vop; VIM; VPL
NMU2	Dyskinetic CP	Dystonia	M	18	GPi; Voa/Vop; VIM; VA
NMU3	Tourette	Comorbid anxiety	M	15	GPi; Voa/Vop; STN; VIM; NA; CMP

^a^Patients were identified from among the population of patients undergoing treatment in the neuromodulation monitoring unit (NMU) at Children's Health Orange County and Children's Hospital of Los Angeles.

CP, cerebral palsy; F, female; M, male; GPi, globus pallidus internus; Voa/Vop, ventral oralis anterior/posterior; STN, subthalamic nucleus; VA, ventral anterior; VPL, ventral posterolateral; NA, nucleus accumbens; CMP, centromedian-parafascicular.

### Experimental design

Motor performance was studied during the execution of figure-eight drawing movements. Tablet software was used to record the two-dimensional coordinates of the fingertip on the tablet (iOS 4.3 operating system; Apple^®^, Cupertino, CA, USA). Each subject was positioned at a distance that allowed them to reach the farthest point on the tablet, which was fixed on the table in portrait orientation. Subjects were asked to follow a 0.3 cm-thick trace on the tablet to draw a figure-eight [15.7 cm (width) × 7.8 cm (height)]. Prior to the start of the experiment, participants were encouraged to be as accurate as possible while tracing the figure-eight at their natural speed. Starting from the upper point of the figure-eight, subjects were requested to move in the mediolateral direction opposite to the arm used to perform the task. The task was performed with their dominant hand. An audible metronome was used to help the subjects maintain approximately consistent speed, and subjects were encouraged to make continuous repeated movements without stopping. The actual speed of movement was subsequently calculated for each cycle based on the fingertip position. The patient with hemidystonia completed five sequences (trials) of 10 figure-eight cycles at average speed of ~18 cycles per minute (cpm). The patient with generalized dystonia completed five trials of 10 cycles at average speed of ~ 8 cpm. The patient with TS completed two trials of 10 cycles at average speed of ~36 cpm.

### Electrophysiological recordings

#### Surgical procedure

Our standard clinical procedure for determining DBS targets includes the implantation of 6–10 temporary AdTech MM16C depth electrodes (Adtech Medical Instrument Corp., Oak Creek, WI, USA) at potential DBS targets (including basal ganglia and thalamic subnuclei), as identified based on clinical criteria in each patient (6). Typical thalamic targets for children include VoaVop, Vim, and basal ganglia such as GPi. The depth electrodes were placed using standard stereotactic procedure for the implantation of DBS electrodes, with the most distal stimulation contact placed at the target location. Electrode location was confirmed by co-registration of preoperative magnetic resonance imaging (MRI) and postoperative computed tomography (CT) scans. Thalamic targeting was confirmed by identification of leads in subnuclei known to have greater or lesser response to median nerve electrical stimulation (9).

#### Deep brain recordings

Recordings were performed during the first 24 to 48 h after clinical implantation of the temporary depth electrodes. Each MM16C electrode lead has a diameter of 1.2 mm and contains 6 low-impedance (1–2 kΩ) ring electrodes with 2-mm height and 5-mm spacing, as well as 10 high-impedance (70–90 kΩ) microwire electrodes (50-μm diameter). The microwire electrodes are arranged in groups of 2 or 3, spaced evenly around the circumference of the electrode shaft, between pairs of ring electrodes. Results reported here are from the microwire electrodes in GPi, VoaVop, and Vim. Therefore, there are either 30 (unilateral) or 60 (bilateral) contacts recorded simultaneously.

**Figure 1:**
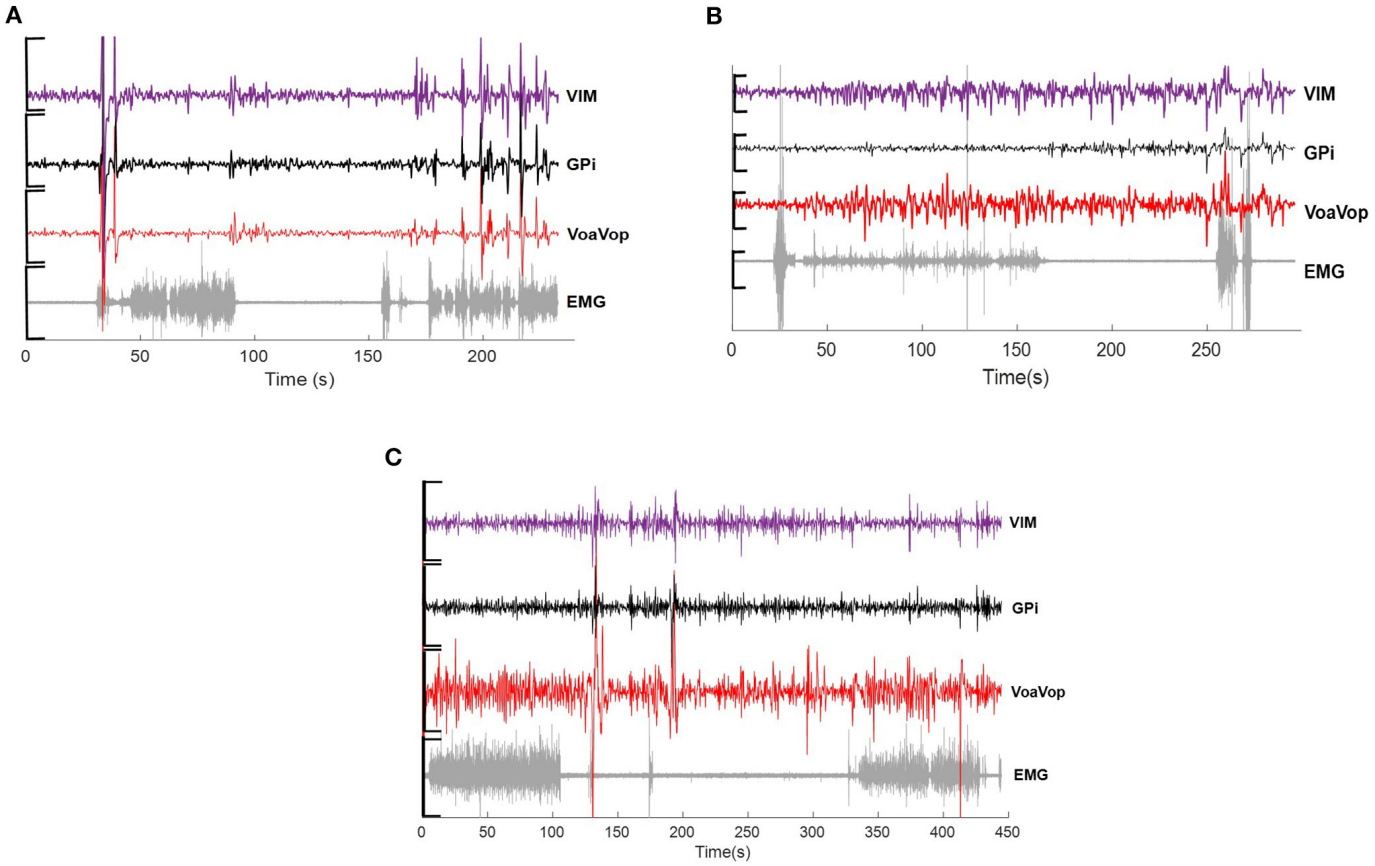
Example of raw data across microwire electrodes placed on Vim (purple), GPi (black), VoaVop (red) and muscle (sEMG, grey), showing changes in activity during drawing task for **(A)** hemidystonia. **(B)** generalized dystonia and **(C)** Tourette patients. Vertical axis: voltage [−5 to 5 uV)]. Horizontal axis: time (s).

The external proximal ends of electrodes were connected to Adtech Cabrio™ connectors, modified to include a custom unity-gain preamplifier for each microwire electrode to reduce noise and motion artifacts. Microwire electrode signals were amplified with a DC-coupled amplifier, sampled at 22 kHz, and digitized by a Tucker-Davis Technologies PZ5M analog-to-digital amplifier connected to an RZ2 digital signal processor. Data were streamed to an RS4 high-speed data storage unit, controlled by Synapse recording software (System3, Tucker-Davis Technologies Inc., Alachua, FL, USA) (Figure 1).

#### Surface EMG recordings

sEMG signals were extracted from six muscles of the upper limb known to contribute to wrist, elbow, and shoulder movements: biceps (Bi), triceps (Tri), anterior deltoid (AntDel), lateral deltoid (LatDel), posterior deltoid (PostDel), and supraspinatus (Supra). Prior to sensor placement, the skin over the muscles and the sensor surface were wiped with isopropyl alcohol pads to reduce electrical impedance at the skin-electrode interface. Six wireless surface sensors (Delsys Trigno, Delsys Inc. Natick, MA, USA) were placed on the patient's muscles and attached with skin adhesive. The sEMG signals were displayed on a monitor to ensure proper placement and signal quality (Figure 1).

### Data analysis

Data were analyzed with Matlab^®^ R2021b software (Mathworks^®^, Natick, MA, USA).

#### Surface-EMG

A non-linear recursive filter based on Bayesian estimation for sEMG signals was applied. Bayesian filtering produces a smooth output that estimates the driving force underlying the EMG signal with low variability, while allowing for the detection of very rapid changes in output. The non-linear filter removes the high-frequency components of sEMG and allows evaluation of the frequency components of the envelope of sEMG activity, thus relating more closely to force produced by the muscles (10).

#### Power spectral density analysis

We used orthogonal multilevel wavelet decomposition (MWD) for signal filtering (11) across all microwire electrodes. Information from GPi, VoaVop, and Vim recordings during the figure-eight drawing with the upper limbs was obtained for all patients. Thus, a total of 24 GPi, 21 VoaVop and 24 Vim contacts were analyzed. In order to detect the frequency related with the motor task, power spectral density (PSD) analysis based on the fast Fourier transform (FFT) (12) was performed for both muscle and brain signals. The sequences for the x- and y-axes in the figure-eight were re-sampled to equalize task duration among subjects, with the horizontal *f*_*x*_ and vertical *f*_*y*_ frequency components sampled at a ratio of 2:1(3):1


(1) $$\begin{array}{r} f_{x}=2^{*}f_{y} \end{array}$$

#### Fitting to gaussian model

The speed to complete the figure-eight varied between trials. To address this issue, task frequency components *f*_*x*_ and *f*_*y*_, for all repetitions in each subject were fitted to a general gaussian model *f*(*x*) of two components:


(2) $$\begin{array}{r} f(x)=a1^{*}exp(-{\left(\frac{x-b1}{c1}\right)}^{2}+a2^{*}\exp\left(-{\left(\frac{x-b2}{c2}\right)}^{2}\right) \end{array}$$

**Figure 2:**
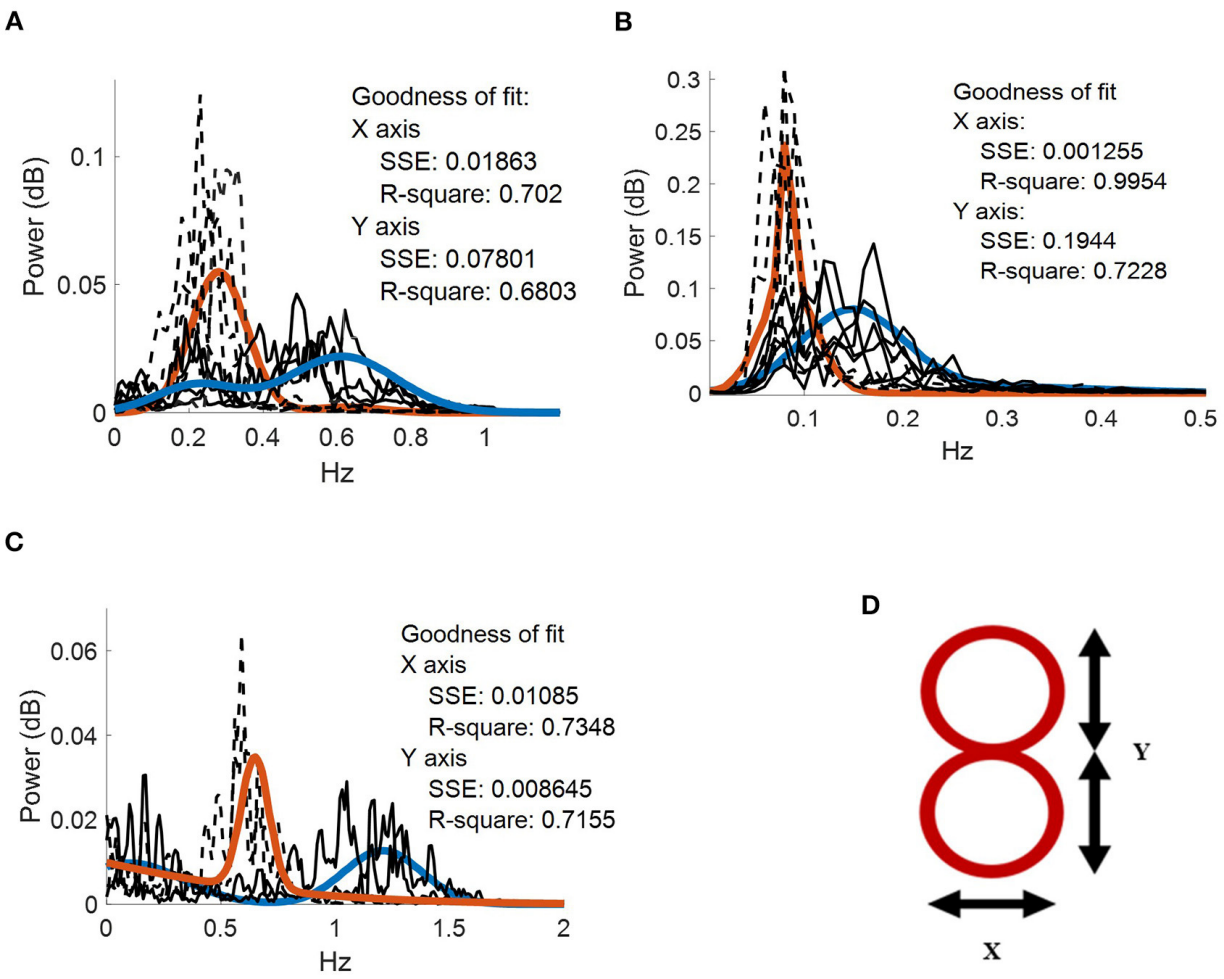
Frequency spectrum of the movement kinematics at the fingertip. Two gaussian fits (blue and orange continuous traces, respectively) applied to *f*_*x*_ (dotted black lines) and *f*_*y*_ (dotted gray lines) task components for. **(A)** five repetitions performed by the patient with hemidystonia. **(B)** five repetitions executed by the patient with generalized dystonia; and **(C)** two repetitions performed by the patient with TS. Vertical axis: power spectra (dB). Horizontal axis: frequency (Hz). **(D)** Representation of the figure-eight drawing trace (0.3-cm thickness).

Where *a*1 represents peak amplitude; *b*1 represents peak centroids; *c*1 and *c*2 are peak width; *x* is normalized by the mean and standard deviation. Thus, all brain and muscle signals can be analyzed under the gaussian curves. The goodness of fit for each frequency component were calculated through R^2^ and sum of square error (SSE) (Figure 2).

A Spearman correlation analysis (ρ = [0–1], *p*-value< 0.001) was also calculated between each kinematic component (*f*_*x*_, *f*_*y*_) and brain or muscle signals to quantify the relationship in terms of frequency for both active and rest conditions.

## Results

### Average of power spectra across repetitions

**Figure 3:**
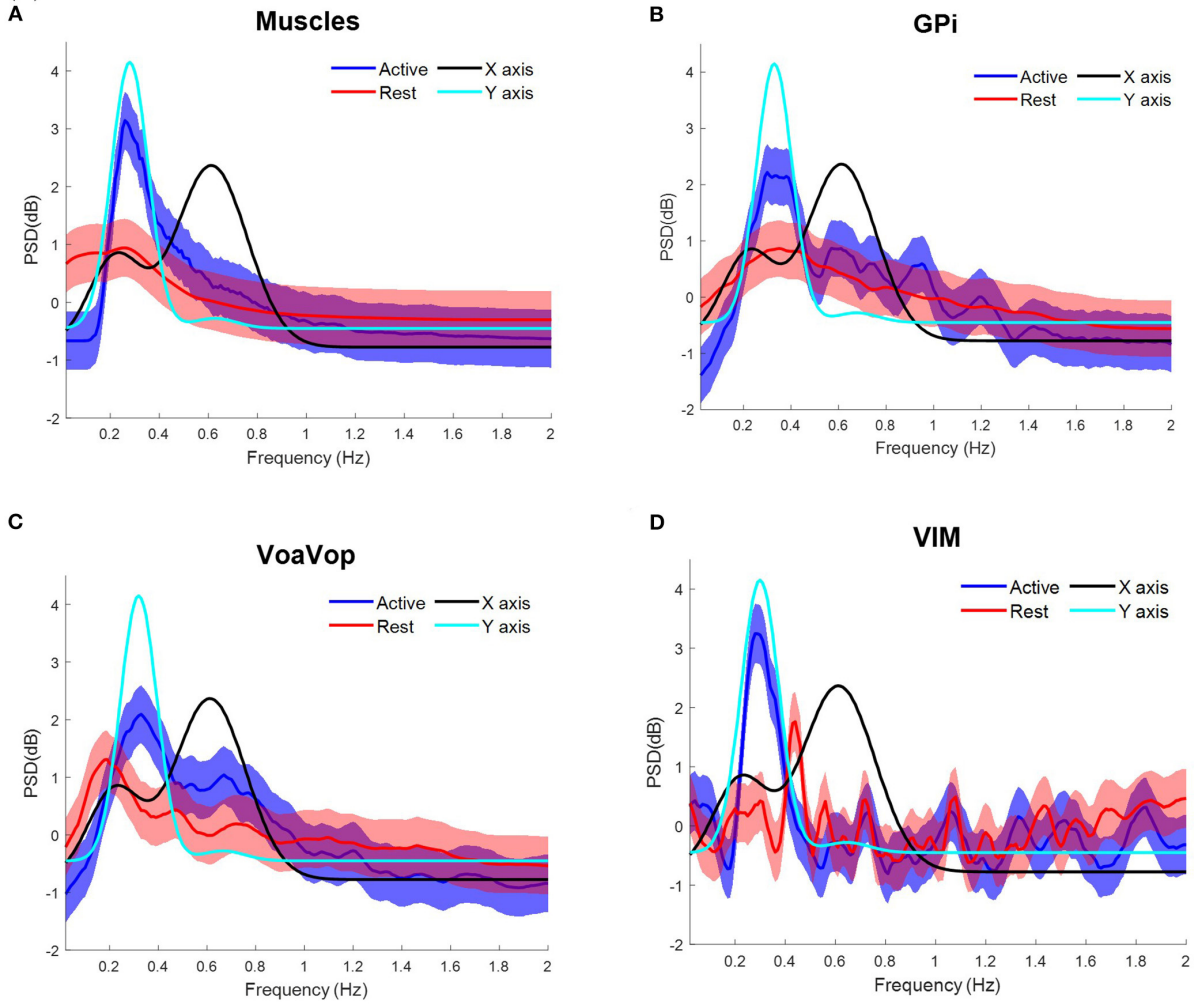
Task frequency components for the patient with hemidystonia. Contralateral muscles and brain signals during the figure-eight drawing task, recorded from multiple surface EMGs and from multiple externalized DBS electrodes. Power spectrum density (PSD) for kinematic data is shown for comparison: *f*_*y*_ component (cyan) and *f*_*x*_ component (black). Grand average and standard deviation across all muscles and all microwire electrode recordings during all repetitions of the figure-eight drawing (blue) and resting (red) conditions. **(A)** Grand average across contralateral muscles. Grand average of left-side brain targets. **(B)** Brain signals from globus pallidus interna (GPi). **(C)** ventral oralis anterior/posterior (VoaVop), **(D)** ventral intermediate (Vim). Vertical axis: power spectra (dB). Horizontal axis: frequency (Hz).

The patient with hemidystonia executed five sets of 10 cycles of figure-eight drawing repetitions at an average speed of ~18 cpm, clearly represented in the PSD of the movement kinematics with peaks matching the *f*_*y*_ components at ~0.3 Hz (Figure 2A). Grand average contralateral muscle activation through all repetitions was strongly associated with the *f*_*y*_ component during the figure-eight drawing (active) as shown in Figure 3A and Supplementary Video 1. The grand average of brain signals across repetitions was also associated with task frequency components during the figure-eight drawing (active), for all targeted brain regions (left brain side) compared with the rest condition (Figure 3). For all brain and muscle signals the PSD peaks were most closely associated with the *f*_*y*_ component.

**Figure 4:**
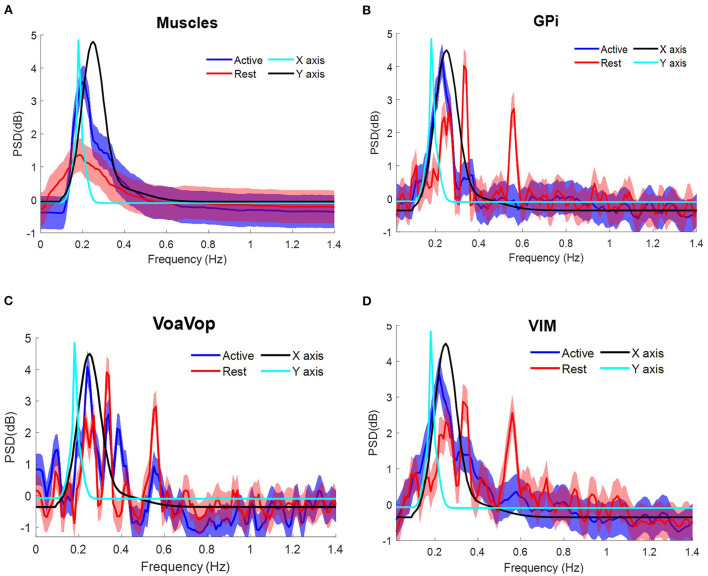
Task frequency components for the patient with generalized dystonia. Contralateral muscles and brain signals during the figure-eight drawing task, recorded from multiple surface EMGs and from multiple externalized DBS electrodes. Power spectrum density (PSD) for kinematic data is shown for comparison: *f*_*y*_ component (cyan) and *f*_*x*_ component (black). Grand average and standard deviation across all muscles and all microwire electrode recordings during all repetitions of the figure-eight drawing (blue) and resting (red) conditions. **(A)** Grand average across contralateral muscles. Grand average of left-side brain targets. **(B)** Brain signals from globus pallidus interna (GPi). **(C)** ventral oralis anterior/posterior (VoaVop), **(D)** ventral intermediate (Vim). Vertical axis: power spectra (dB). Horizontal axis: frequency (Hz).

The patient with generalized dystonia executed the five sets of 10 cycles of figure-eight repetitions at an average speed of ~8 cpm, clearly represented in the PSD with peaks matching the *f*_*x*_ and *f*_*y*_components at ~0.12 Hz. Grand average contralateral muscle activation was strongly associated with the kinematic component during the figure-eight drawing (active), (Figure 4A). Similarly, brain signals during the figure-eight drawing (active) showed peaks matching the *f*_*x*_ component (Figure 4, Supplementary Video 2).

**Figure 5:**
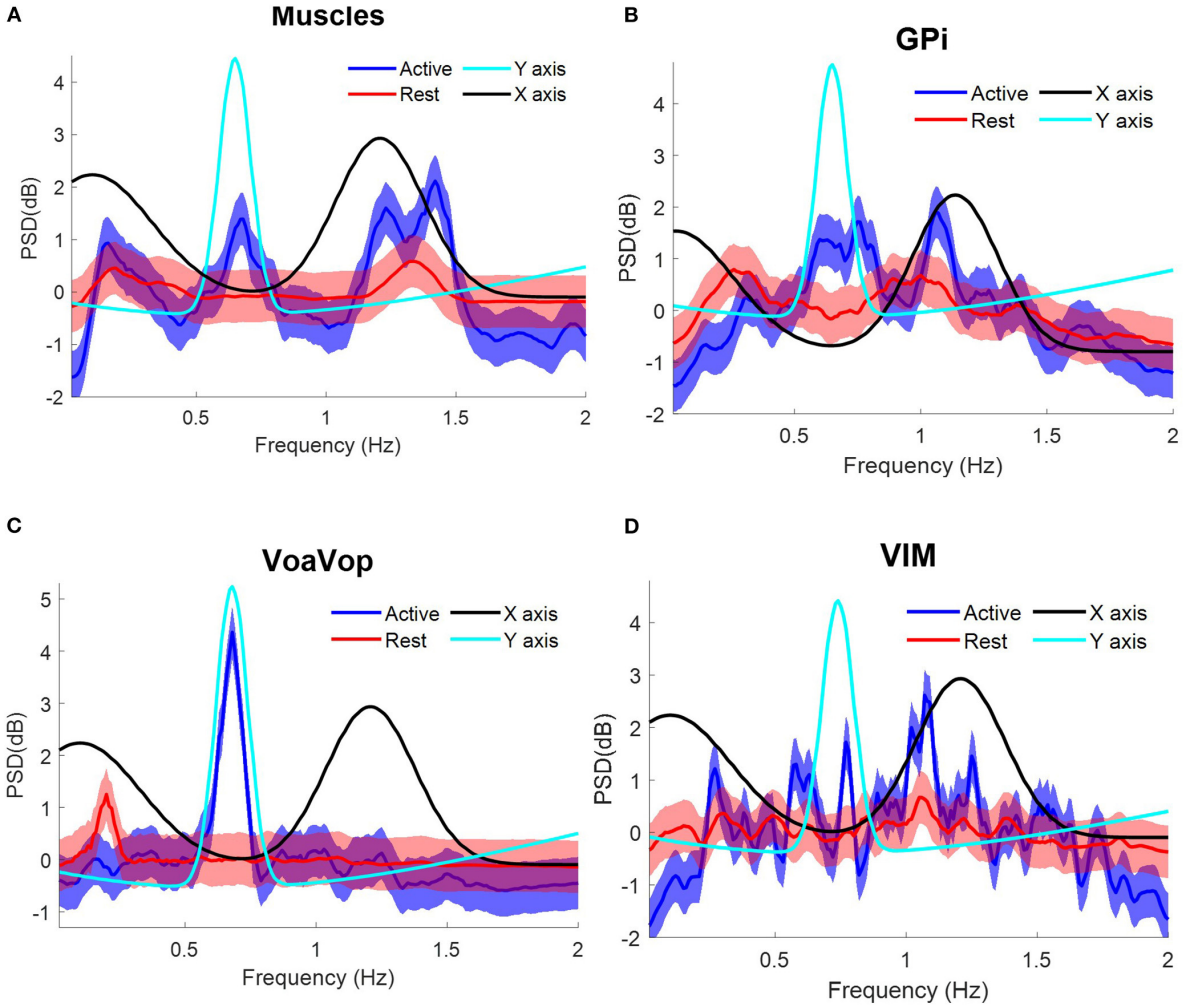
Task frequency components for the patient with Tourette syndrome. Contralateral muscles and brain signals during the figure-eight drawing task, recorded from multiple surface EMGs and from multiple externalized DBS electrodes. Power spectrum density (PSD) for kinematic data is shown for comparison: *f*_*y*_ component (cyan) and *f*_*x*_ component (black). Grand average and standard deviation across all muscles and all microwire electrode recordings during all repetitions of the figure-eight drawing (blue) and resting (red) conditions. **(A)** Grand average across contralateral muscles. Grand average of left-side brain targets. **(B)** Brain signals from globus pallidus interna (GPi). **(C)** ventral oralis anterior/posterior (VoaVop), **(D)** ventral intermediate (Vim). Vertical axis: power spectra (dB). Horizontal axis: frequency (Hz).

The same frequency analysis was computed for the patient with TS through two sets of 10 cycles at average speed ~36 cpm, matching with the *f*_*y*_ component at ~0.6 Hz as represented in the PSD. Following the same data analysis, the grand average for measured muscle activation showed peaks associated with both *f*_*x*_ and *f*_*y*_ components (Figure 5A). Grand average brain signals for GPi and Vim were associated with the task frequencies (active) more closely in GPi than in Vim. In contrast to Vim and GPi, VoaVop showed a peak matching with the *f*_*y*_ component (Figure 5, Supplementary Video 3).

### Relationship between brain and muscle recordings during the cyclic drawing task

**Figure 6:**
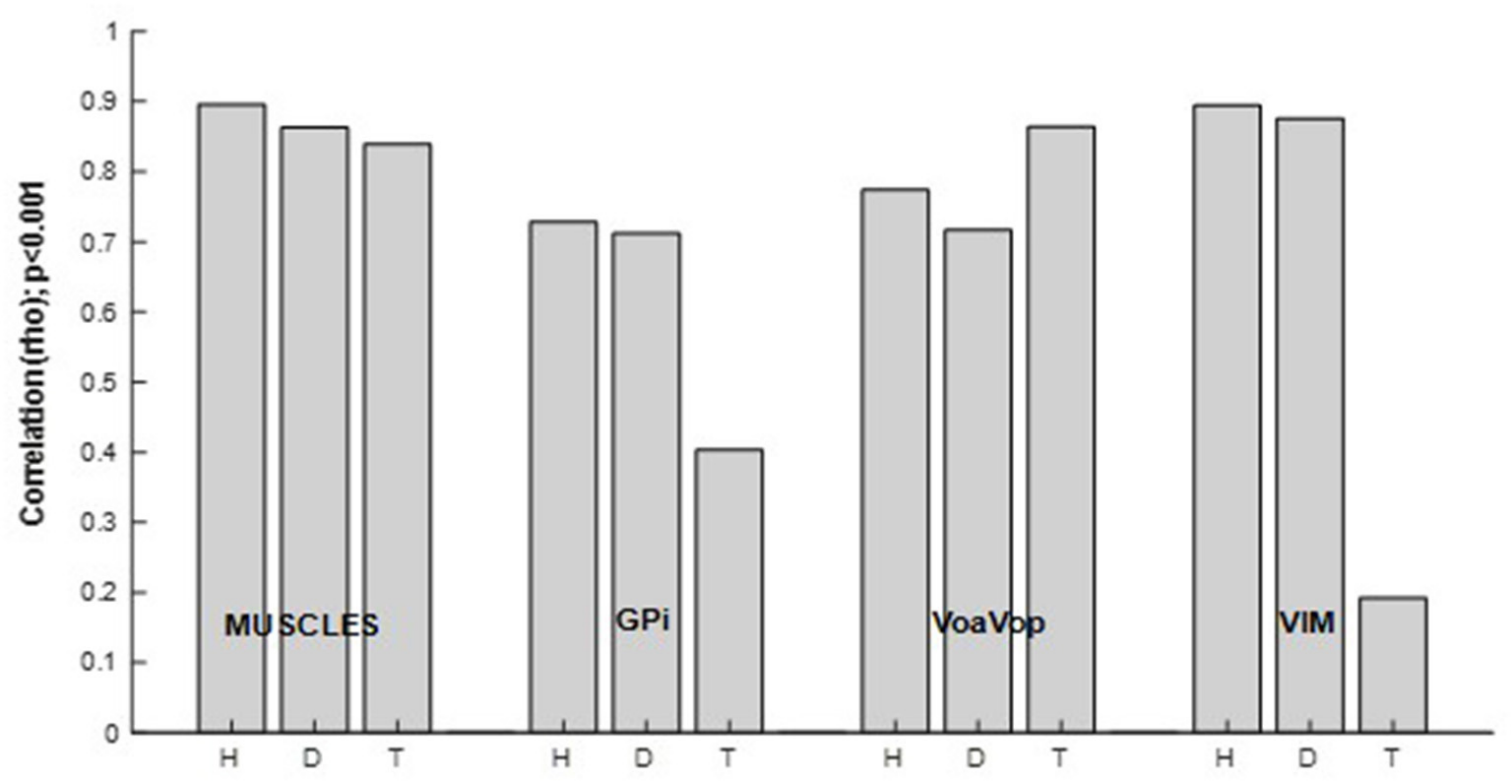
Spearman correlation (ρ [0–1], *p* < 0.001) between power spectrum of the kinematics of movement, and the muscle and brain signals power spectra. Results are shown separately for the patients with hemidystonia (H), generalized dystonia (D) and Tourette syndrome (T). Spearman correlation for the combined *f*_*x*_ and *f*_*y*_ components and all involved muscles or brain signals across all repetitions.

To determine the relationship between all muscles and kinematic components during the figure-eight drawing task (active) and rest conditions, Spearman analysis was performed (Figure 6). Spearman' correlations showed significant results (*p* < 0.001) for the active conditions, in contrast to the rest conditions (*p* > 0.001), thus the active conditions are only represented in this analysis. All patients showed high correlation indices (ρ > 0.8, *p* < 0.001) for the muscle signals. The same calculation was used to study the relationship between brain signals and kinematic components. As we predicted, motor thalamic nuclei (VoaVop) showed high correlations with task execution (ρ > 0.7, *p* < 0.001), while basal ganglia (GPi) showed lower correlations with the task (ρ < 0.7, *p* < 0.001), across all patients. We also expected to see a strong correlation between the sensory thalamic subnuclei (Vim) and the task components, because Vim is expected to encode proprioceptive sensory signals transmitted through cerebellum. Therefore, a strong correlation (ρ > 0.8, *p* < 0.001) was observed for the patients with generalized dystonia and hemidystonia, in contrast to the Tourette syndrome patient.

## Discussion

The most striking feature of our results is the significantly increased task-related activity in GPi and Vim in the patients with dystonia and hemidystonia compared to the patient with Tourette Syndrome (TS) (Figure 6). There was no difference in task-related activity in VoaVop, or in the muscles. While the analysis is limited to the power spectral density and data were obtained from only 1 subject in each category, this result nevertheless suggests that increased task-related activity in both GPi and Vim could be a feature of dystonic movement. This result is consistent with a role of both basal ganglia and cerebellum in the pathogenesis of dystonia. For example, hemidystonia did not show correlation between the task frequency component and VPL (Supplementary Figure S1A). Or the generalized dystonia patient, which did not show any correlation between task frequency component and VA nuclei (Supplementary Figure S1B).

It is also consistent with our observation that both GPi and Vim can be effective targets for amelioration of symptoms in a subset of children (VoaVop is also an effective target in some children, possibly because it is in the output pathway from GPi) (13–15).

Since dystonia is activated by attempts at task performance, this increased task-related activity could reflect increased neural activity responsible for dystonic muscle contractions or overflow. An important caveat is that thalamic nuclei receive strong inputs from motor cortical areas, and thus we cannot be certain whether the increased task-related activity in thalamus arises from basal ganglia and cerebellum or could instead be due to increased task-related inputs from cortex.

GPi has been strongly implicated in the pathophysiology of dystonia, both because of symptoms resulting from injury, and the ability to ameliorate symptoms in a subset of patients using lesions or stimulation in GPi. The cerebellum has been conjectured to be one potential contributor to dystonia (15–17), but it is not known whether this would result in increased signals transmitted through the Vim nucleus of the thalamus (18). Our results suggest that both areas are likely to be involved, although whether they are causal, compensatory, or associated cannot be determined from these data.

Figure 6 shows strong correlations between Vim and task components for hemidystonia and generalized dystonia. This finding may be explained by the role of the cerebellum (16, 17, 19) in the pathophysiology of dystonia. Vim receives input from cerebellum (18) and there could be an alteration of cerebellar input (20) due to abnormal proprioceptive sensory signals arising from the dystonic movement. Further studies will be needed to determine the significance and generalizability of this finding. Nevertheless, our results indicate an alteration in the transfer of information from the peripheral system, providing further evidence for Vim as a possible target for DBS in dystonia (21, 22).

It is interesting to compare the levels of task-related spectral power in the various regions. As expected, muscles have the highest correlation. In the two dystonic subjects, Vim has almost as high a correlation, which could represent either a causative role in the dystonic components of the movement, or increased compensation for abnormal movements, or increased proprioceptive feedback. In all 3 subjects, high levels of VoaVop activation may represent the role of thalamus in the control of both healthy and dystonic movement. In other words, just as motor cortex is involved in both healthy and pathological movement, VoaVop may be an important modulator that both receives input from motor cortex and contributes to activity in motor cortex, whether this activity is related to healthy or to involuntary movement. Slightly decreased activity in VoaVop in the two subjects with dystonia (*p* < 0.05) is consistent with the slightly increased activity in GPi (*p* < 0.05) in these two subjects compared to TS, since the projections from GPi to VoaVop are inhibitory (23).

Both subjects with dystonia moved slowly compared to the subject with TS, and therefore we were not able to compare the magnitude of task-unrelated components between subjects. With a larger cohort, relative magnitudes of task-unrelated components could potentially be correlated against the magnitude of task-related and task-unrelated components in the brain signals.

Previous neurophysiological studies are consistent with the theory that during voluntary movement the basal ganglia are responsible for the focused selection of the desired motor pattern and for the inhibition of undesired and competing movements (24, 25). Under this theory, decreased focusing of inhibitory output from GPi would permit disinhibition of involuntary movements in the thalamus and cortex. Our finding of increased GPi activity in the two subjects with dystonia could represent broadening of output predicted by decreased focusing, although it is not clear why the total output would increase. We cannot exclude the alternative possibility that increased GPi activity is compensatory, perhaps an attempt to reduce involuntary movement by increasing the focused or unfocused inhibitory output to thalamus. Because we look only at the magnitude of the PSD and not the phase, we cannot determine whether the timing of the GPi outputs could be incorrect, perhaps inhibiting when disinhibition would be expected, and disinhibiting when inhibition would be expected. Interestingly, our results appear to be inconsistent with the “rate model” of basal ganglia activity, in which dystonia is conjectured to be associated with thalamo-cortical disinhibition due to decreased output from GPi (26).

The results presented above do not allow for definite conclusions regarding the pathophysiology, or whether there might be other mechanisms that can cause the motor disorder. Moreover, our data do not allow us to compare patients with movement disorders to completely healthy subjects. Nevertheless, the patient with TS could be interpreted as a non-dystonic brain since the motor coordination has not been altered and the patient has no symptoms consistent with dystonia.

One limitation of this study is the limited sample size; nonetheless, the number of task repetitions and the number of muscle measurements are sufficient to predict results in the study population. The findings described above may also provide the foundation for future studies that use the cyclic drawing task, in contrast to free voluntary movements, to show how deep brain structures respond to movements in a specific manner, thus providing consistent and reliable frequency patterns for future investigation.

In summary, our results show that task-related signals are present in GPi, VoaVop, and Vim in both dystonic and non-dystonic subjects, but that the GPi and Vim nuclei have higher levels of task-related activity in the two subjects with dystonia. Therapeutic response to deep brain stimulation in these regions supports the hypothesis that this activity is at least in part responsible for abnormal movement. This is consistent with a role of both basal ganglia and cerebellum in the pathogenesis of dystonia. Further studies will be necessary to determine use of the cyclic drawing task as a tool for neurophysiological investigation in subjects with movement disorders.

## Data availability statement

The raw data supporting the conclusions of this article will be made available by the authors, without undue reservation.

## Ethics statement

The studies involving human participants were reviewed and approved by Health Insurance Portability and Accountability Act (HIPAA). Written informed consent to participate in this study was provided by the participants' legal guardian/next of kin.

## Author contributions

TS conceived and supervised the experiment. EA acquired the data. EH-M conducted the experiment and analyzed the data. ML and AR implanted the DBS electrodes. EH-M and TS interpreted the results and wrote the manuscript. All authors contributed to the final manuscript.

## Funding

This work was supported by funding from the Cerebral Palsy Alliance Research Foundation Inc., (PG02518). Research reported in this publication is supported by CHOC.

## Conflict of interest

The authors declare that the research was conducted in the absence of any commercial or financial relationships that could be construed as a potential conflict of interest.

## Publisher's note

All claims expressed in this article are solely those of the authors and do not necessarily represent those of their affiliated organizations, or those of the publisher, the editors and the reviewers. Any product that may be evaluated in this article, or claim that may be made by its manufacturer, is not guaranteed or endorsed by the publisher.
