# Supplementary material for: Increased movement-related signals in both basal ganglia and cerebellar output pathways in two children with dystonia
Source: Front Neurol. 2022 Sep 9;13:989340. doi: 10.3389/fneur.2022.989340 (PMC9500435; doi:10.3389/fneur.2022.989340)
Supplement: Supplementary file 4 [file Data_Sheet_1.PDF]

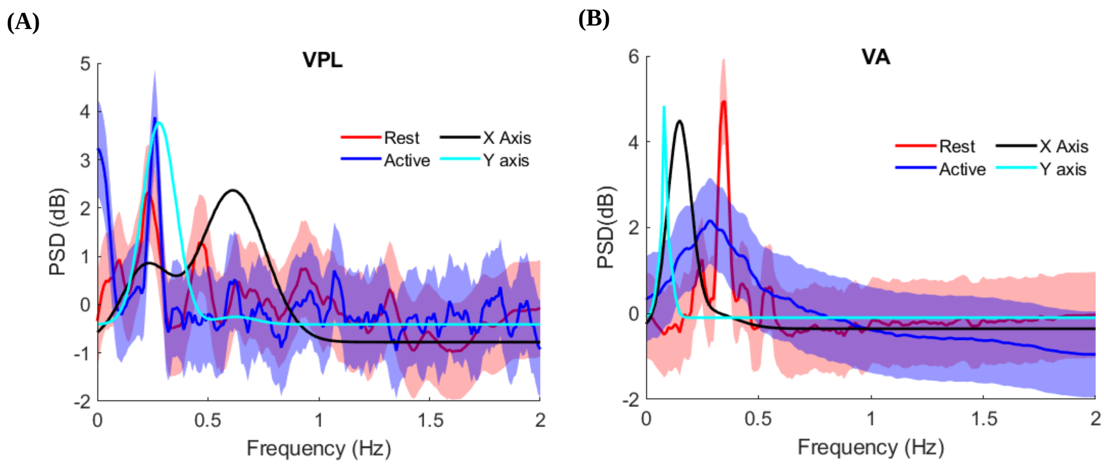

Figure S1. Task frequency components for the patient with (A) hemidystonia and (B) generalized dystonia. Contralateral brain signals during the figure-eight drawing task, recorded from (A) ventral posterolateral and (B) ventral anterior thalamus subnuclei. Power spectrum density (PSD) for kinematic data is shown for comparison: f_y component (cyan) and f_x component (black). Grand average and standard deviation across all microwire electrode recordings during all repetitions of the figure-eight drawing (blue) and resting (red) conditions. Vertical axis: power spectra (dB). Horizontal axis: frequency (Hz). VPL did not show significative correlations with task execution ($\rho<0.7$, $p<0.001$). VA did not show correlations with the task frequency components ($\rho<0.7$, $p<0.001$), according to the Spearman' correlations.
